# A Study on Dictionary Selection in Compressive Sensing for ECG Signals Compression and Classification

**DOI:** 10.3390/bios12030146

**Published:** 2022-02-27

**Authors:** Monica Fira, Hariton-Nicolae Costin, Liviu Goraș

**Affiliations:** 1 Institute of Computer Science, Romanian Academy, 700481 Iasi, Romania; monica.fira@iit.academiaromana-is.ro (M.F.); lgoras@etti.tuiasi.ro (L.G.); 2 Faculty of Electronics, Telecomunications & Information Technology, Gheorghe Asachi Technical University of Iasi, 700050 Iasi, Romania

**Keywords:** compressed sensing, ECG signal, reconstruction dictionaries, projection matrices, signal classifications

## Abstract

The paper proposes a comparative analysis of the projection matrices and dictionaries used for compressive sensing (CS) of electrocardiographic signals (ECG), highlighting the compromises between the complexity of preprocessing and the accuracy of reconstruction. Starting from the basic notions of CS theory, this paper proposes the construction of dictionaries (constructed directly by cardiac patterns with R-waves, centered or not-centered) specific to the application and the results of their testing. Several types of projection matrices are also analyzed and discussed. The reconstructed signals are analyzed quantitatively and qualitatively by standard distortion measures and by the classification of the reconstructed signals. We used a k-nearest neighbors (KNN) classifier to evaluate the reconstructed models. The KNN module was trained with the models from the mega-dictionary used in the classification block and tested with the models reconstructed with class-specific dictionaries. In addition to the KNN classifier, a neural network was used to test the reconstructed signals. The neural network was a multilayer perceptron (MLP). Moreover, the results are compared with those obtained with other compression methods, and ours proved to be superior.

## 1. Introduction

Compressed sensing (CS) is a method of signals acquisition and processing based on the fact that sparse or rare signals can be reconstructed from a relatively small number of projections on a set of random signals [1]. This technique is relatively new compared to classical techniques, so in recent years, a large number of papers on implementation, applicability, advantages and the pertinence to dedicated types of signals have been published [2,3,4,5,6,7,8,9,10,11,12].

Many of the papers that address CS focus on how to build specific dictionaries for signal reconstruction [13,14,15,16,17,18,19,20,21,22,23,24,25,26]. In the case of the ECG signal, due to its particularities, namely, the quasi-periodicity of the P, Q, R and S waves and the preservation of their shapes, many of the methods proposed in the literature focus on the advantages offered by these features specific to the ECG signal [27,28,29,30,31,32,33,34,35,36,37]. Thus, a large part of the methods proposed regarding CS of ECG signals aim at building dictionaries specific to these signals. In many cases, building these dictionaries involves a preprocessing step with or without signal segmentation, with or without QRS wave alignment. Another aspect regarding CS applied to ECG signals is the optimization of the compression matrix.

In the following lines, we will briefly present some specific ECG methods proposed in the literature over the past years, which contain results similar to the methods we presented in this paper, except for the fact of using patient-specific dictionaries or involving updating the dictionary when there are changes in the ECG signal. In general, there is a big inconvenience in the situation of using such a system in practice, because it involves resubmitting the dictionary and necessary calculations in real time to see if the dictionary is good or needs to be updated. All these calculations imply additional hardware needs, which can make the method less practical in real-time acquisition situations. On the other hand, our approach is based on the use of non-modified patient-specific dictionaries or pathology-specific dictionaries; these are established once and updating can be done less frequently than in other techniques and does not require real-time decisions.

In one paper [33], the presented method uses an over complete wavelet dictionary, a dictionary that is later reduced due to a training phase. In addition, it is proposed to align the beats according to the position of the R-peak. This alignment aims to exploit the different scaling characteristics of ECG waves in the wavelet dictionary optimization process. Three different methods are tested for dictionary optimization. It should be mentioned that this optimized dictionary is specific to the patient and for its construction, the first 5 minutes of registration are taken. For acquisition, the authors use a matrix optimized for the ECG signal to be acquired through CS. The use of an optimized compression matrix leads to improved results, but has the disadvantage that once this matrix is changed it must be sent together with the compressed ECG signal. That means both the compressed ECG signal and the compression matrix must be sent to restore the ECG signal.

Another approach is presented in [34], where the quasi-periodic character of the ECG signal is used to detect similarities between ECG pulses and to transmit segments that show dissimilarities normally, without compression. This approach is proposed because abnormal frames, which could be signs of heart disease, are not similar to normal frames. Thus, only the ECG segments considered normal are transmitted by CS, the rest being transmitted normally. Once it is determined, whether the heartbeat is acquired normally or by CS, a quantization step follows and then a Huffman compression. These two steps lead to improved compression results. A critical point in the method is the correct detection of normal vs. abnormal beats, because this automated detection is debatable in the light of the fact that normality or abnormality is determined by a cardiologist and the accuracy of the acquisition should not be influenced by this decision.

In paper [35], the authors also used CS associated to dictionaries built specifically for the ECG signal, thus using the dictionary learning technique to construct a better sparsifying basis to improve the compression ratio. Moreover, the authors consider the change of ECG signal characteristics and propose a physiological variation detection technique and a low-complexity dictionary refreshing algorithm to update the dictionary from time to time when the current dictionary is no longer suitable for the patient.

Many papers in the CS field focus on optimizing the measurement matrix, i.e., the matrix is used in the acquisition stage or on optimizing the necessary calculations in this stage by arranging this matrix in a way that allows easy hardware implementation of the necessary calculations. In practical implementations, the simple random or Bernoulli matrix may have the inconvenience of the required number of operations. Thus, in paper [36], the authors propose an optimized algorithm for collecting the compressed ECG signal, based on the proposed optimization of a deterministic binary block diagonal matrix. The blocks, which make up the diagonal of the matrix, are identical and contain m = N/M elements each, where M and N, respectively, represent the number of rows and columns.

In paper [37], a new method of compressive sampling of ECG signals is presented, which is based on the idea of building the compression matrix adapted to the frame of the ECG signal to be compressed. Thus, a circulating matrix is proposed, containing zeros and ones, obtained by quantizing (with 1-bit resolution) the size of the ECG signal. The detection matrix adapted in this way guarantees that the significant portions of the waveform of the compressed ECG signal are in fact contained in the compressed version. In this way, a more precise reconstruction is guaranteed in relation to the methods already available in the literature. For the reconstruction stage, the acquisition matrix is then used in combination with a modified wavelet dictionary, which also allows the reconstruction of the signal deviation for each processed frame. The big disadvantage of the method is that whenever the acquisition matrix has to be updated, it has to be sent to the receivers and for reconstruction we have to know each frame with which matrix was collected.

In this paper, we propose a detailed comparative study of two different approaches regarding the possibility of compressed sensing specific ECG signals. This study considers several acquisition techniques/projection matrices used in the acquisition stage and several dictionaries used in the ECG signal reconstruction stage. We will also analyze the effect of preprocessing on the results.

Broadly speaking, we analyze and discuss two CS approaches dedicated to ECG signals, namely:

1. An approach that is based on the direct CS obtaining of the signal, without preprocessing it prior acquiring the projections. This “genuine” CS we call patient-specific classical compressed sensing (PSCCS), since the dictionary is constructed from a patient’s initial signals.
2. A variant that involves a module of pre-processing and segmentation of the ECG signal. This stage aims at improving the scatter and recoverability of the ECG signal. In this additional stage of preprocessing, the ECG signal results in the rhythmicization of the ECG signal and divides it into cardiac cycles—hereinafter referred to as cardiac patterns compressed sensing (CPCS). Now the acquired signals and atoms of dictionary are segmented heartbeats pre-processed without or with the R-waves centered.

For both approaches from above, we will analyze several projection matrices, namely, matrices with random independent and identically distributed (i.i.d.) elements taken from the Gaussian or Bernoulli distribution and project matrices optimized for the particular dictionary used in the reconstruction. To optimize the projection matrix, the method presented in [7] will be used.

Furthermore, we will pay special attention to the way the dictionary is built. We will also present the advantages and disadvantages of each and the choice of the method that depends on the available hardware and software resources.

The paper is organized as follows: Section 2 is dedicated to the types of sampling vectors, projection matrices and dictionary construction methods. Section 3 presents the CS methods dedicated to ECG signals. Section 4 shows the results obtained. In Section 5 the results from the previous section are compared and in Section 6 conclusions are drawn.

## 2. Compressed Sensed Overview

Traditionally, signals are acquired according to the sampling theorem [8] that states that an f_0_-bandlimited signal can be recovered from its samples if the sampling frequency is at least 2 f_0_, i.e., twice the highest frequency of the signal spectrum. Thus, in a time window W, an f_0_-bandlimited analog signal can be represented by N = 2 f_0_ W samples equally spaced at T = 1/2 f_0_, i.e., as a vector belonging to the space R^N^. Such a signal can be alternatively defined by using any complete set of orthogonal functions in R^N^. In fact, sampling is nothing else than taking projections (scalar products) on the elements of the canonical basis. In the general case the signal can be reconstructed from its projections on N orthogonal (or only linear independent) elements in RN the canonical basis being the most frequently used. However, in practice, there are cases in which a signal can be reconstructed from fewer samples or projections on an appropriate set of signals, compared to the number prescribed by the sampling theorem. This is possible since the samples contain unnecessary information, and thus, these signals can be compressed and recovered using projections and previous known information. An example would be the class of sparse or rare signals [9,10,11] that allow a representation based on a small number of elements/atoms in RN. In signal processing literature, the name “k-sparse” denotes signals that can be reconstructed by means of k of elements of RN, the most significant situation being that in which *k<<N*. A discrete signal or vector $x\in R^{N}$ is k-sparse if there exists a base $\Psi =\{\Psi_{i},i=1,\ldots ,N\}$ in R^N^ so that most of the elements $\alpha =\{\alpha_{i},i=1,\ldots ,N\}$ of its representation in that basis, x=Ψα, are zero. Alternatively, they can be approximately zero, so that the signal can be represented accurate enough with the k’s largest terms $\alpha_{i}$ from its expansion with respect to that basis. The CS concept is based on theory that a k-sparse signal, i.e., a signal that can be compressed into a base (or, more general, dictionary) Ψ can be recuperated with very good quality from a number *m* of the order of scale m=O(klog(N/k)) of non-adaptive linear projections on a set of vectors Φ, which are not comprehensible with the first, i.e., their elements cannot be used for a compressed representation of any $\Psi_{i},i=1,\ldots ,N$. Therefore, for obtaining the measurement signal instead of measuring the *N* components of the signal in the canonic base, a number of m (k<m<<N) linear projections on the elements of the matrix $\Phi^{N*m}$ are acquired:

(1) y=Φx=ΦΨα=Θα

where the measurement noise was not taken into account. If we use as a projection matrix (noted with Φ) a matrix with dimensions *mxN*, with *m < N*, then it means that we will make a number of m measurements, each measurement of size *N*. That is, the vectors on which *x* is projected represent the rows of the projection matrix.

The main idea regarding (1) is that, because m<N, the rebuilding of the original signal cannot be realized, but only under the compressibility hypothesis. It has been shown that if Φ and Ψ satisfy certain conditions, the original vector α can be obtained as the unique result to the optimization problem:

(2) $$\hat{\alpha }=\arg\min{\left\|\alpha \right\|}_{l_{0}}\;subject\;\;to\;y=\Phi \Psi \alpha ,$$

where *l_0_* is the (pseudo)norm consisting of the number of nonzero entries of α.

The reconstructed signal has the form:

(3) $$\hat{x}=\Psi \hat{\alpha }$$

corresponding to the sparsest representation of *y* in terms of the dictionary ΦΨ. To circumvent the problems of combinatorial nature and noise effect in the case of almost sparse signals, two directions evolved:

(i) seeking for a suboptimal solution of problem (2) and
(ii) using the Basis Pursuit (BP) procedure [1] that consists of replacing $l_{0}$ with $l_{1}$ minimization, by resolving problem (4) instead of the initial one:
  (4) $$\hat{\alpha }=\arg_{\alpha }\min{\left\|\alpha \right\|}_{l1}\;subject\;to\;y=\Phi \Psi \alpha$$

Let us stress the fact that although pure sparse signals (built of exactly *k*<<*N* atoms from a specified dictionary) are difficult to find, conventional results are valid for signals that are “almost sparse” (which can be built of *k*<<*N* non-negligible atoms) with respect to dictionaries that can be overcomplete (contain more atoms than their intrinsic dimension), as in the case of some classes of biomedical signals. Taking into consideration this fact, it has been found useful to adapt the theory of CS to the field of processing ECG and electroencephalographic (EEG) signals [2,3,4] as well as for applications [5] such as compression, transmission, reconstruction of ECG signals, ECG filtering and monitoring [6,27,30,31,32].

For a better understanding of the algorithm, in the following we present a pseudocode summary.

INPUTS: ORIGINAL SIGNAL = x

Acquisition Stage:

Step 1: Compute random measurements

y= Φx, where Φ is a MxN matrix of random independent and identically distributed (i.i.d.) entries.

Reconstruction Stage:

Step 2: Compute α coefficients using L1 minimization

$\hat{\alpha }=\arg_{\alpha }\min{\left\|\alpha \right\|}_{l1}\;subject\;to\;y=\Phi \Psi \alpha$

Step 3: Reconstruct original signal $x^{\prime }$

$x^{\prime }=\;\alpha \Psi$

OUTPUTS: RECONSTRUCTED SIGNAL = $x^{\prime }$

## 3. Sample Vectors, Projection Matrices and Dictionaries

Here, we briefly show several ways of segmentation, a couple of projection matrices, as well as some several ways of building various types of dictionaries specific to the ECG signal. Depending on the chosen CS method, the way of building the dictionary which is used to reconstruct the ECG signal is different.

### 3.1. Sample Vectors

First of all, let us mention that we will refer to ECG signals with a sampling frequency according to the Nyquist–Shannon sampling theorem of 360 Hz and 300 (or 301 for case with R-wave centered) samples/vector, respectively. Each vector is projected on a number of random vectors with identic size and the obtained values are utilized for recovering through a dictionary.

In the simplest way, the first 300 samples of the ECG signal set up the first vector; then, the succeeding 300 samples form the second vector, etc. The place of the R-wave can be anywhere in a vector or it may be missing sometimes, which is, obviously, not desirable.

In order to take advantage of the cyclicity of the ECG signal and of the changes produced on the ECG signal in case of some diseases, we proposed some modified acquisition techniques that requires preprocessing [13,14,15,16]. Thus, samples of the ECG signal are stored in a buffer zone and a series of preprocessing can be performed on these stored signals. The R-waves can be detected, and based on them, the ECG signal can be segmented into cardiac patterns. A cardiac pattern is delimited by the halves of the RR intervals of two adjacent intervals and re-sampled by interpolation so that the pattern has a fixed number of 301 samples. The above segmentation and preprocessing technique contain simple calculations and, as will be presented, notably increases results for the compression and reconstruction processes.

Starting from the method described above, an improvement of the cardiac model can be obtained by centering the R-wave on sample 151. Thus, a resampling to the left of the R-wave will be performed and another resampling to the right of the R-wave and the final cardiac model will have 301 samples with the R-wave centered. This alignment of the R-wave can be a reversible process, provided that the reduction/stretch ratio from left to right is known. To make a much clearer picture of the re-sampling and alignment effect, we provide in the following examples of unfocused (misaligned) heartbeats and the same cycles prepared to be aligned. These segments constitute atoms in the dictionary or preprocessed sample vectors.

**Figure 1:**
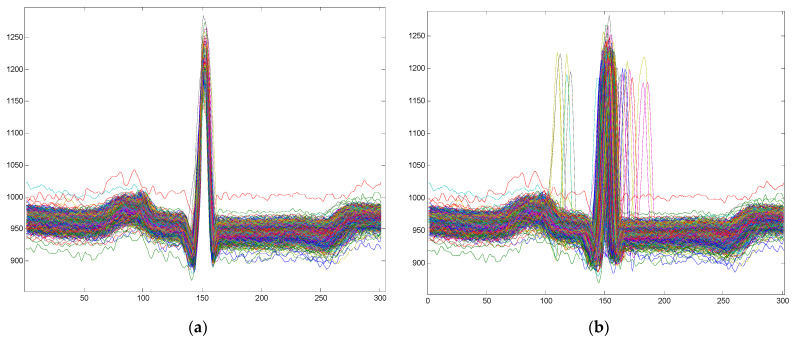
Examples of cardiac patterns obtained by centered or non-centered R-wave: (**a**) Cardiac patterns with a centered R-wave; (**b**) Cardiac patterns without a centered R-wave.

Figure 1 shows examples of cardiac models with and without a centered R-wave.

In conclusion, the sampling vectors and the atoms of the dictionary can be: (i) un-processed or pre-processed through segmentation and resampling or (ii) segmented and resampled with a centered R-wave.

### 3.2. Projection Matrices

A key element in the CS method is the projection matrix for the acquisition of the ECG signal. The reconstruction quality of the ECG signal is considerably decided by the kind of the matrix used in the compression stage [7,9,10,13].

Moreover, the number of random vectors (and respectively the number of calculated scalar products) considered is based on the tolerated tradeoff between the compression ratio and the reconstruction error: thus, the compression ratio is directly related to the reconstruction error.

In Section 3, we will analyze and determine which is a stop ratio and we will determine in the case of our ECG signals how many projections we need for a good ECG compression.

In the following, we use and discuss three types of projection matrices.

- As so far shown in Introduction and CS theory, projecting on a matrix Φ results in a system. A simple approach is to use as Φ a random matrix with i.i.d. normal elements. Nevertheless, this matrix has a higher Restricted Isometry Property (RIP) constant and, thus, it is inappropriate for reconstruction [7].
- Another possibility is to build a projection matrix specific to the dictionary used in the reconstruction phase. Thus, we can define such a matrix as a product of the random matrix and the transposition of a square matrix containing an arbitrary selection of N dictionary atoms [7]. In this way, the reconstruction errors will be smaller. In the tables with results, we denote this matrix with “Random * Dict †”.
- A third possibility of projection matrix analyzed in this paper is the Bernoulli type matrix built only of elements of 0s and 1s, with symmetric distribution (half of the inputs of a row are created with the Bernoulli distribution and the other half reversing the first half) [14]. The advantage of this matrix is the low computational complexity, and thus, saving of IT resources.

In this paper, we examine the consequence of these three types of projection matrices on various dictionaries.

### 3.3. Dictionaries

Using standard Discrete Cosinus/Sinus Transform (DCT/DST), Wavelet or other typical dictionaries is not always the best choice if we are referring to ECG signal reconstruction errors [15]. Thus, we will analyze the use of dictionaries dedicated to ECG signals, dictionaries that can be specific to the patient, specific to the pathology or universal. The way dictionaries are built is closely related to the segmentation methods of the ECG signals presented above. Thus, concerning the preprocessing stage, we used dictionaries with three types of atoms: (1) Unprocessed (patient-specific only) and processed atoms; (2) Segmented atoms; (3) Segmented plus R-wave centered. The last two types contain either patient-specific beats, or normal beats and/or seven types of pathological beats.

#### 3.3.1. Patient-Specific Dictionaries

In order to build patient-specific dictionaries, we used the first minutes of each patient’s record and then the rest of the ECG signal was used for testing. Thus, the atoms represent ECG segments of size 300, successive segments of vectors, without any processing. In our studies, such dictionaries were constructed (only) from the first few minutes of the patient’s records (patient-specific dictionary), the atoms being further used for CS with various projection matrices.

In order to maintain uniformity in the size of the dictionaries, we chose to build patient-specific dictionaries of 700 atoms, each atom having a size of 300. A size of 300 for atoms was determined considering the sampling frequency (360 Hz) and the average beat frequency heart rate (~70 beats/min for normal patients). In this way, the dictionary is actually a matrix with a size of 300 × 700. We highlight that the atoms of the dictionary were aleatory sequences of the ECG recording, and therefore, the R-wave can appear anywhere in the 300 samples or even be missing (not a happy case).

We note that besides the simplicity of the ECG signal segmentation method, another advantage is the capture of the specificity of the patient’s ECG particularities in the moment the recording has started.

An improved version of the method is to preprocess the ECG segments to build the dictionary. Thus, segmentation can be performed by detecting heartbeats (i.e., R-waves) and then the R-wave centers. Therefore, patient-specific dictionaries can be constructed without or with preprocessing for R-wave alignment. However, in all cases, the first portion of an ECG recording is used to construct the dictionary, while the rest of the signal (the unused part in the dictionary) was used in the testing techniques.

The next two types of dictionaries contain only atoms obtained through segmentation, normalized to 301 elements with or without a centered R-wave.

#### 3.3.2. Universal Mega-Dictionaries

The mega-dictionary used consists of 1472 atoms (i.e., 184 beats from each of the 8 classes discussed, 7 pathological and the normal beat class). Depending on the preprocessing tested, the atoms of the dictionary may or may not have a centered R-wave.

#### 3.3.3. Pathology-Specific Dictionaries

When the reconstruction stage considers the pathologic class that the cardiac beat belongs to, a particular or specific dictionary has been constructed for each pathological class. Because the ECG recordings include heartbeats from several pathological classes, we tested the variant in which, for each pathological class, we made a specific dictionary. Thus, analyzing 7 pathological classes and the normal class, we built 8 dictionaries, each with 700 atoms specific to each class. Atoms may or may not have a centered R-wave. Thus, we note that the number of atoms in each of the dictionaries is higher than the number of atoms related to a certain pathology contained in the mega-dictionary.

## 4. Proposed Methods for Dictionary-Based ECG Compression

In the Introduction, we talked about the presentation of two totally different methods of CS specific to ECG signals, but both have in common the need of building specific dictionaries. However, the use of ECG signal characteristics and how to build dictionaries differ remarkably.

Thus, the PSCCS method is based on ECG signal specific features of each patient, while CPCS on the cyclical patterns of the heartbeat.

In the next subsection, we present two methods for CS of ECG signals with some dissimilarity associated to the projection matrices.

### 4.1. Patient-Specific Classical Compressed Sensing—PSCCS

**Figure 2:**
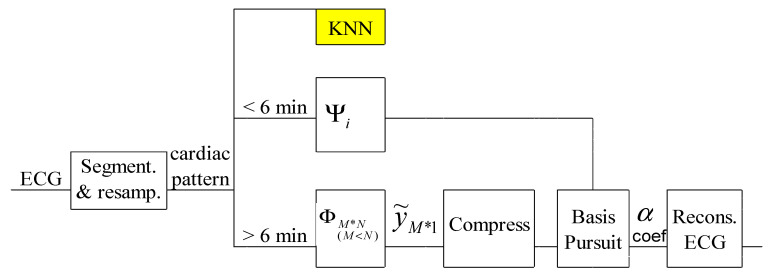
Principle of the PSCCS method.

A first variant of compressed acquisition of the ECG signal is presented in Figure 2. It can be implemented even on hardware system and involves the compressed collection of the ECG signal using the CS technique and a patient-specific dictionary together with the Basis Pursuit technique [14].

In this method, the compression of the ECG signal involves the classic use of the CS technique, without any additional signal processing. The advantage of the method is that it speculates on the specific features of the patient. Another advantage is the reduced complexity equal to that of the traditional CS algorithm. The particularity of this procedure is the need for a classic 6-minute ECG acquisition to build the dictionary. In order to obtain improved results, the dictionary can be upgraded in case of long recordings or in case the patient has undergone changes on the ECG signal from one recording to another.

### 4.2. Cardiac Patterns Compressed Sensing—CPCS

Below, we present a different approach from the classic CS, which involves a preprocessing stage used both for segmentation of the ECG signal for compressed acquisition and for building useful dictionaries in the signal reconstruction stage.

**Figure 3:**
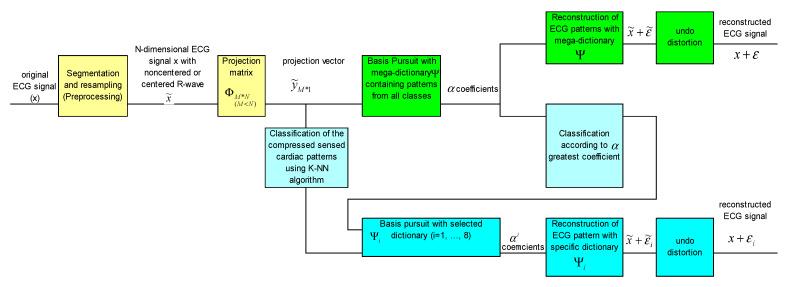
Block diagram of the CSCP method, using the mega-dictionary and/or a pathology-specific dictionary.

Figure 3 shows the block diagram of the method. As we can see, at the level of the reconstruction stage there are two approaches, namely, a way of reconstruction using a mega-dictionary or another variant in which dictionaries specific to pathologies are used. The first two operations are common to both approaches and are colored in yellow in the block diagram.

The upper branch of the block scheme, colored in green, is for the version with the universal mega-dictionary and the lower part of the figure, colored in blue, is for the version with dictionaries specific to pathologies.

In the case of reconstruction with dictionaries specific to pathologies, it is necessary to know the pathological class to which each cardiac pattern belongs. Therefore, it is necessary to classify the heartbeats. One option is to use a KNN classifier or any other classifier trained with various compressed beats [15,17]. Another option for classifying the heartbeats is a first reconstruction with the mega-dictionary on the upper branch of Figure 3 and the analysis of alpha coefficients corresponding to the mega-dictionary, i.e., the pathological class associated with the heartbeats is the same as the class in which the atom in the mega-dictionary with the highest coefficient belongs at reconstruction with the BP algorithm. Once the pathological class is established, the final reconstruction will be performed with the dictionary specific to that pathology [16].

For the classification of the ECG pattern and the establishment of the dictionary with which the signal will be reconstructed, the KNN classifier trained with the compressed version of the heartbeat from the universal mega-dictionary can be used.

Thus, a first step is to establish the class of the pattern. For this, we will use the KNN classifier based on the highest coefficient corresponding to the mega-dictionary, shown in light blue in Figure 3. Once the membership class is established, the Basis Pursuit algorithm together with the calculation of α coefficients necessary for the reconstruction of the ECG pattern are used. In addition, the almost insignificant distortions due to the centering of the R-wave can be improved by means of the knowledge about the original location of the R-wave.

### 4.3. Acceptance of the Compression Methods

To evaluate the compression and reconstruction performances, we assess the distortion between the original and the reconstructed signals by standard *PRD* and *PRDN* measures. Most ECG compression algorithms in the literature evaluate the errors using the percentage root-mean-square difference (*PRD*) measure and its normalized version, *PRDN*, defined as:

$$PRD\%=100\sqrt{\frac{\sum_{n=1}^{N}{\left(x(n)-\tilde{x}(n)\right)}^{2}}{\sum_{n=1}^{N}x^{2}(n)}}$$

and:

$$PRDN\%=100\sqrt{\frac{\sum_{n=1}^{N}{\left(x(n)-\tilde{x}(n)\right)}^{2}}{\sum_{n=1}^{N}{\left(x(n)-\bar{x}\right)}^{2}}}$$

where x(n) and $\tilde{x}(n)$ are the samples of the original and the reconstructed signals, respectively, $\bar{x}$ is the mean value of the original signal and *N* is the length of the window over which the *PRD* is calculated.

For the evaluation of the compression, we used the compression rate (*CR*) defined as the ratio between the number of bits needed to represent the original and the compressed signal:

$$CR=\frac{b_{orig}}{b_{comp}}$$

where $b_{orig}$ and $b_{comp}$ represent the number of the bits required for the original and compressed signals, respectively.

We also used an alternative measure defined in [19], the Quality Score (*QS*), which is the ratio between the *CR* and the *PRD*:

$$QS=\frac{CR}{PRD}.$$

In addition to the quantitative measure related to the reconstruction of ECG signals, we also used a qualitative evaluation of the signals by classifying them. For classification, we used the KNN classifier. Thus, in the CPCS method version with a pathology-specific dictionary, in order to estimate the signal classification ratio in one of the eight possible classes, we used a KNN classifier to evaluate the reconstructed models. We mention that the KNN was trained with the models from the mega-dictionary used in the classification block (models that were not subjected to compression with the known class for each atom) and tested with the models reconstructed with class-specific dictionaries.

In addition to the KNN classifier, a neural network was used to test the reconstructed signals. The neural network was a multilayer perceptron (MLP) with 10 neurons in the hidden layer with backpropagation gradient descent for training.

However, the final verdict on the fidelity and clinical acceptability of the reconstructed signal should be validated by visual inspection by the cardiologist.

## 5. Experimental Results

In this study, we used 24 ECG recordings from the MIT-BIH Arrhythmia database acquired at a sampling frequency of 360 Hz, with 11 bits/sample [18]. Besides the ECG signals, the database also includes annotation files containing the index of the R-wave and the class to which each ECG pattern belongs.

In the CPCS method, we used the annotation databases in the preprocessing step (segmentation of cardiac cycles and forming of dictionaries) and in the reconstructed signal validation phase (KNN classifier-training stage).

The PSCCS technique used only the ECG signals from the MIT-BIH database, without requiring additional knowledge (ECG annotated files).

### 5.1. Results for the Patient-Specific Classical Compressed Sensing (PSCCS) Method

To test the PSCCS procedure, we used several compression ratios, namely, 4:1, 10:1 and 15:1. We also used several types of projection matrices (Bernoulli, Gaussian distribution random and dictionary specifics). The data used are 24 records from the MIT-BIH Database. In Table 1, we present the average results for 24 ECG records.

In addition to the average results reported for the MIT-BIH database, a number of authors reported the results for record no. 117 (in Table 2), which is why we will report these results as well.

**Figure 4:**
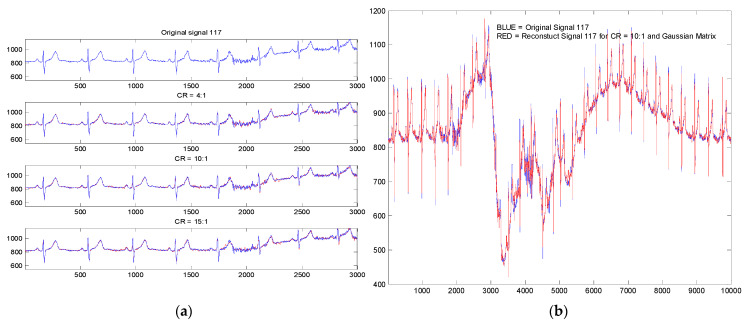
Original (blue) and reconstruct (red) ECG signal with PSCCS method (registration no. 117): (**a**) for CR 4:1, 10:1 and 15:1 with a Bernoulli projection matrix; (**b**) for CR 10:1 with random projection (Gaussian distribution).

In Figure 4a, we present a part of the registration no. 117 in the initial version and its version reconstructed following the compression of 4:1, 10:1 and 15:1 for the application of a Bernoulli type projection matrix. It is observed that for CR = 15:1, especially in the noisy region (sample from 2000 to 2200), there are some visible reconstruction differences due to this noise. There are no significant differences in the rest of the signal.

In Figure 4b, we also present from the recording 117 an original ECG signal segment and its variant reconstructed subject to a CR = 10:1 (for random projection matrix with Gaussian distribution). The segment shown is the segment with the highest noise in the entire recording. In this way, we wanted to highlight the robustness of the method to noise and artifacts due to the patient’s movement and breathing.

The results obtained on 14 ECG signals, for a compression ratio of 15:1, for centered and non-centered R-wave are shown in Table 3. We used the KNN and MLP algorithm for the evaluation by classification.

The KNN and MLP classifiers were trained with normal and abnormal heart beats evenly distributed on both classes. The beats used to train the classifier were extracted from the dictionary constructed for the compressed acquisition. In this case, the classification was on two classes, normal or abnormal, and it did not follow the seven pathological classes.

The advantage of the KNN classifier is the simplicity of the calculations, this classifier assuming only the calculation of some Euclidean distances. In the case of MLP networks, the calculations are more complex, but the results are better compared to the KNN classifier.

### 5.2. Results for the Cardiac Patterns Compressed Sensing (CPCS) Method

#### 5.2.1. Universal Mega-Dictionary

For the construction of a mega-dictionary, from all the 24 ECG recordings, we randomly chose 184 patterns from the 8 cardiac classes, thus obtaining a dictionary with 1472 patterns with the size 1472 × 301.

The testing was performed on 200 patterns from each class, chosen at random from the 24 records, with the mention that special attention was paid to random choice, namely, the models used to build the dictionary could no longer be used for testing.

Table 4 shows the average results obtained on all 24 records, with R-wave alignment and centering and without R-wave centering, for all the projection matrices presented.

#### 5.2.2. Pathology-Specific Dictionaries

Each of the eight pathology-specific dictionaries is made up of 700 atoms that actually represent patterns with or without a centered R-waves. Dictionaries are matrices of size 700 × 301.

For testing, we used a number of 2000 cardiac patterns chosen at random from the 24 records with the mention that the patterns used for testing are different from those used for training (see Table 5).

In this variant, with dictionaries specific to the pathological class, in the reconstruction stage, it is necessary to identify the class to which the pattern belongs. The reconstruction results are strongly influenced by the correctness of establishing the pathological class to which the model belongs. Thus, for patterns classification, a KNN type classifier will be used or it will be made based on the highest alpha coefficient. Once the pathological class is established, the Basis Pursuit algorithm, the dictionary specific to that pathology and the projection matrix will be used for reconstruction.

Thus, using the classification of patterns based on the highest alpha coefficient in the mega-dictionary version, a pattern classification rate of 88.75% is obtained [16]. Using the KNN classifier with training on 1472 compressed cardiac patterns (uniformly distributed in the eight classes), a classification rate of 93.77% is obtained [15].

**Figure 5:**
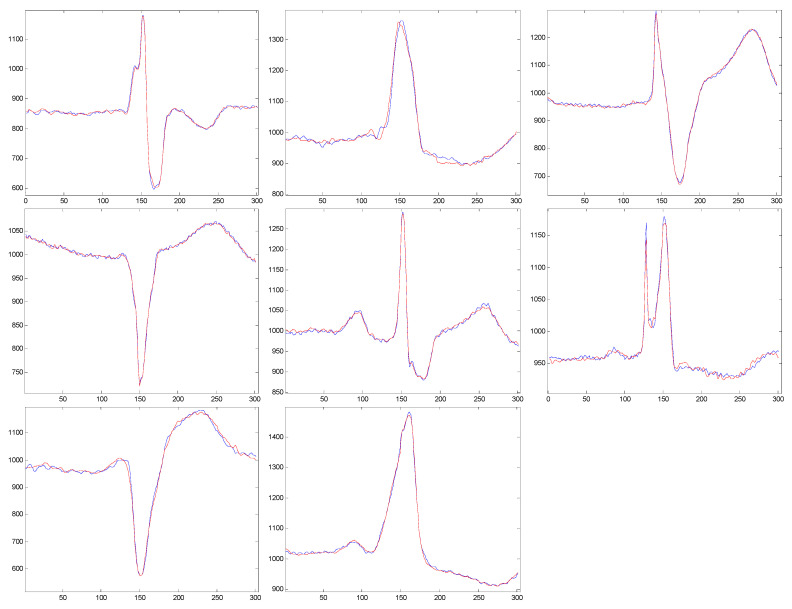
Original and reconstructed signals with pathology-specific dictionaries.

In Figure 5, we present examples of reconstructed cardiac beats for every pathology class.

Qualitative estimation of reconstructed signals based on classification. In addition to the quantitative measures of the distortions between the original and reconstructed ECG signals, for a further verification of the quality of the proposed compression scheme, we performed a classification of reconstituted models with the KNN algorithm. The classifier was trained with the atoms from the mega-dictionary. A first check of the method is to test the performance of the KNN classifier, and for this, we initially tested the original models (i.e., the uncompressed models that we used to test the compression scheme). For these patterns, we obtained a classification rate of 93.75%. The results presented below are obtained on the reconstructed patterns [28].

- Classifying the patterns reconstructed with the mega-dictionary (with patterns out of all classes) yielded an accuracy of 92.5%.
- Classifying the patterns reconstructed with the class-specific dictionaries provided an accuracy of 95.5%.

In addition to KNN, an MLP classifier was also tested. This second classification aims to strengthen the correctness of the idea of testing the reconstructed patterns from a qualitative point of view. This test is based on a classifier and is needed to compare the results obtained with these two different classifiers. Thus, there is a slight and almost insignificant improvement of the classification rate in the case of MLP compared to KNN. However, in practical implementations, the MLP classifier should be chosen according to the available hardware resources. Table 6 shows obtained results for dictionaries with a centered R-wave.

It is known that in a classification process, especially when it applies to several classes, special attention must be assigned to the confusion matrix, to see if the classification is uniform on all classes or only certain classes are detected. For this we have exemplified in Table 7 a confusion matrix for the classification variant with a mega-dictionary. It can be seen that the classification rate is evenly distributed over all eight classes.

#### 5.2.3. Patient-Specific Dictionaries

The patient-specific dictionaries were constructed from the patient’s first 700 heartbeats, and preprocessed as previously described (i.e., with or without R-wave alignment). Thus, the dictionary is made up of 700 atoms, each of size 300, i.e., it is actually a matrix of size 301 × 700. This method has the advantage of speculating quasi-periodicity and the particular characteristics of the ECG signal of a particular patient. Table 8 shows average results for 24 ECG recordings for the CSCP method and it can be seen that the best results are obtained if we refer to QS.

**Figure 6:**
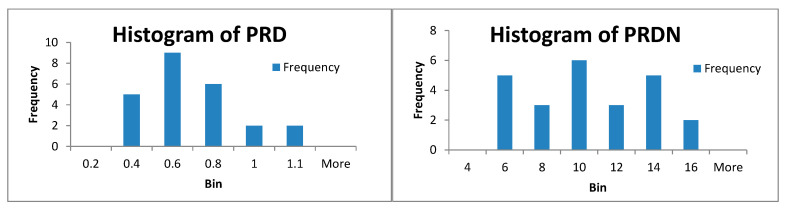
Histogram of PRD and PRDN for 24 ECG records for the CSCP method with a patient-specific dictionary with projection matrix by type of Gaussian distribution Random * Dict †.

Because our results are generally obtained by mediating the results obtained by processing 24 records from MIT-BIH Arrhythmia database, we present in Figure 6 the histograms of PRD and PRDN, respectively, for the method of CS with patient-specific dictionaries with a centered R-wave and projection matrix by type Gaussian distribution Random * Dict †. For this case, PRD_average = 0.51 and PRDN_average = 9 (see Table 8).

## 6. Discussions

In Table 9, we resume the results previously presented for the two analyzed methods, for a CR = 15:1 with all investigated projection matrices and with all discussed reconstruction and preprocessing dictionaries. We marked in bold the best results obtained on QS (Quality Score) for each method.

It can be seen that the best QS result is obtained for dictionary specific to the patient in which the R-wave is centered and a projection matrix is optimized to the dictionary. In addition, it has been also found that in all cases optimization of dictionaries improves the results. Moreover, it has also been observed that preprocessing improves the results, namely, for PSCCS (i.e., without preprocessing) for CR = 15:1 the best QS equals 15.46, i.e., almost half of the value obtained with CPCS with a patient-specific centered R-wave dictionary when QS = 29.13.

It should be noted that any preprocessing means hardware resources and choosing a method with preprocessing means additional hardware resources. However, we must mention that the detection of the QRS complex and the R-wave is a problem that can be implemented in real time in the Matlab^®^ environment, an example of implementation being even available in Help Matlab^®^ [29].

Table 10 shows the average results on the 24 records and for the 117 records obtained by other authors.

We note that Mamaghanian in [22] presents a classical CS compression method followed by Huffman coding, the final CR being higher due to the additional Huffman compression. For a more accurate comparison, we must compare our results with those obtained by Mamaghanian before Huffman compression. Additionally, the same author uses in [22] the compression ratio defined as:

$$CR=\frac{b_{orig}-b_{comp}}{b_{orig}}*100,$$

which is not the same as ours and gives a very different gamut of values compared with ours.

The results we obtained with the proposed method are compared in Table 11 with the results of other compression methods in the literature.

## 7. Conclusions

The results presented in this paper reveal several interesting aspects, as follows.

It has been revealed that the first stage of the CS method, i.e., the signal acquisition part, based on the projection matrices, has only a relatively small influence on the decompression or classification results.

On the other hand, for the second stage, namely signal reconstruction, the dictionary used for reconstruction of the compressed sensed ECG signals has an essential role in obtaining good results. Therefore, depending on the application targeted with the used CS technique, namely, Holter monitoring or recorded ECG signal classification, a dictionary that leads to optimal final results can be selected.

Thus, in a Holter monitoring application, where the ECG signal is recorded for 24 h from the same patient, one can choose the Patient-Specific Classical Compressed Sensing (PSCCS) method. By analyzing the first minutes of the recording, a dictionary specific to the patient will be built, and then it will be used to reconstruct the ECG segments of interest to the specialist.

Otherwise, if the CS-based application aims at classifying heartbeats for ECG monitoring or abnormality identification applications, the Cardiac Patterns Compressed Sensing (CPCS) method will be chosen, where each pathological heart beat class will be associated to a specific dictionary.

The above discussed methods are primarily based on waveform segmentation (cardiac beats) with no preprocessing. Yet, depending on the available hardware resources and the time constraints in which the application should run, the results can be significantly improved by centering the R-wave using ECG preprocessing i.e., segmented cardiac patterns with a centered R-wave.

This choice is related to the idea that any ECG signal preprocessing leads to higher hardware requirements and slowdowns in the acquisition and reconstruction processes over time. However, these aspects can be easily dealt with, aiming at better results. However, we must mention that the detection of the QRS complex and the R-wave is a problem that can be implemented in real time in the Matlab^®^ environment, with an example of implementation even being available in Help Matlab^®^.

## Figures and Tables

**Table 1:** Average results for 24 ECG records processed with the PSCCS method.

Projection Matrix and Its Size	CR	AVG. PRD	AVG. PRDN	QS
Gaussian distribution Random * Dict ^†^	4:1	0.31	6.47	12.9
Bernoulli with 0 and 1 (75 × 300)	4:1	0.41	7.96	9.75
Gaussian distribution Random (75 × 300)	4:1	0.43	8.56	9.30
Gaussian distribution Random * Dict ^†^	10:1	0.67	13.42	14.92
Bernoulli with 0 and 1 (30 × 300)	10:1	0.81	15.49	12.34
Gaussian distribution Random (30 × 300)	10:1	0.82	16.48	12.19
Gaussian distribution Random * Dict ^†^	15:1	0.97	21.31	15.46
Bernoulli with 0 and 1 (20 × 300)	15:1	1.31	23.28	11.45
Gaussian distribution Random (20 × 300)	15:1	1.13	25.37	13.27

**Table 2:** Results for the 117 records processed with the PSCCS method.

Projection Matrix and Its Size	CR	AVG. PRD	AVG. PRDN	QS
Gaussian distribution Random * Dict ^†^	4:1	0.19	4.69	21.05
Bernoulli with 0 and 1 (75 × 300)	4:1	0.40	7.20	10
Gaussian distribution Random (75 × 300)	4:1	0.45	8.12	8.88
Gaussian distribution Random * Dict ^†^	10:1	0.45	11.19	22.22
Bernoulli with 0 and 1 (30 × 300)	10:1	0.70	12.67	14.28
Gaussian distribution Random (30 × 300)	10:1	0.73	13.21	13.69
Gaussian distribution Random * Dict ^†^	15:1	0.63	15.61	23.80
Bernoulli with 0 and 1 (20 × 300)	15:1	0.96	17.28	15.62
Gaussian distribution Random (20 × 300)	15:1	1.01	18.24	14.85

**Table 3:** Average results for 14 ECG records with the PSCCS method.

Projection Matrix and Its Size	CR	AVG. PRD	AVG. PRDN	Classif. Rate with KNN	Classif. Rate with MLP
**Patient-specific dictionary with a non-centered R-wave**					
Gaussian distribution Random * Dict ^†^ (20 × 301)	15:1	0.78	11.98	92.24%	93.7%
Bernoulli with 0 and 1 (20 × 301)	15:1	0.94	16.06	84.71%	86.2%
Gaussian distribution Random (20 × 301)	15:1	0.82	13.82	91.14%	93.4%
**Patient-specific dictionary with a centered R-wave**					
Gaussian distribution Random * Dict ^†^ (20 × 301)	15:1	0.51	9	93.41%	95.2%
Bernoulli with 0 and 1 (20 × 301)	15:1	0.71	12.4	88.06%	90.3%
Gaussian distribution Random (20 × 301)	15:1	0.72	12.51	89.70%	91.6%

**Table 4:** Average results for 24 ECG records processed with the CSCP method with the mega-dictionary.

Projection matrix and Its Size	CR	AVG. PRD	AVG. PRDN	QS
**Mega-dictionary with a non-centered R-waves**				
Gaussian distribution Random * Dict ^†^	15:1	0.88	13.67	17.04
Bernoulli with 0 and 1 (20 × 301)	15:1	1.44	21.43	10.41
Gaussian distribution Random (20 × 301)	15:1	1.62	24.33	9.25
**Mega-dictionary with a centered R-waves**				
Gaussian distribution Random * Dict ^†^	15:1	0.67	9.99	22.38
Bernoulli with 0 and 1 (20 × 301)	15:1	1.08	15.47	13.88
Gaussian distribution Random (20 × 301)	15:1	1.19	17.18	12.60

**Table 5:** Average results for 24 ECG records for CSCP method with a specific dictionary and classification based on the largest coefficient of the sparsest decomposition for the mega-dictionary.

Projection Matrix and Its Size	CR	AVG. PRD	AVG. PRDN	QS
**Pathological specific dictionaries with a non-centered R-wave**				
Gaussian distribution Random * Dict ^†^	15:1	0.77	11.76	19.48
Bernoulli with 0 and 1 (20 × 301)	15:1	1.23	17.90	12.19
Gaussian distribution Random (20 × 301)	15:1	1.37	20.25	10.94
**Pathological specific dictionaries with a centered R-wave**				
Gaussian distribution Random * Dict ^†^	15:1	0.62	6.14	24.19
Bernoulli with 0 and 1 (20 × 301)	15:1	0.97	13.93	15.46
Gaussian distribution Random (20 × 301)	15:1	1.04	14.80	14.42

**Table 6:** Results summary for dictionaries with a centered R-wave.

Dictionary with a Centered R-Wave	Compression Rate	AVG. PRD	AVG. PRDN	KNN Classif. Rate	MLP Classif. Rate
mega-dictionary	10:1	0.47	6.24	93.2%	93.8%
mega-dictionary	15:1	0.67	9.99	92.5%	93.1%
specific dictionaries	10:1	0.43	6.02	95.2%	96%
specific dictionaries	15:1	0.62	6.14	95.5%	96.2%
KNN classification results with original patterns				95.5%	96%
PRDN and KNN classification rate for the case with correct identification (100%) of the specific dictionary		0.55	8.53	93%	93.7%

**Table 7:** Confusion matrix for KNN classification of the reconstructed patterns with a mega-dictionary.

	Class1	Class2	Class3	Class4	Class5	Class6	Class7	Class8
**class1**	**90**	10	0	0	0	0	0	0
**class2**	20	**70**	0	0	0	10	0	0
**class3**	0	0	**100**	0	0	0	0	0
**class4**	0	0	0	**100**	0	0	0	0
**class5**	0	0	0	0	**100**	0	0	0
**class6**	0	0	0	0	0	**100**	0	0
**class7**	0	0	0	0	0	0	**100**	0
**class8**	0	10	0	0	0	0	10	**80**

**Table 8:** Average results for 24 ECG Records for the CSCP method with a patient-specific dictionary built from the first 700 cardiac cycles.

Projection Matrix and Its Size	CR	AVG. PRD	AVG. PRDN	QS
**Patient-specific dictionary with a non-centered R-wave**				
Gaussian distribution Random * Dict ^†^	15:1	0.78	11.98	19.23
Bernoulli with 0 and 1 (20 × 301)	15:1	0.94	16.06	15.87
Gaussian distribution Random (20 × 301)	15:1	0.82	13.82	18.29
**Patient-specific dictionary with a centered R-wave**				
Gaussian distribution Random * Dict ^†^	15:1	**0.51**	**9**	29.13
Bernoulli with 0 and 1 (20 × 301)	15:1	0.71	12.4	20.98
Gaussian distribution Random (20 × 301)	15:1	0.72	12.51	20.59

**Table 9:** Results summary for CR = 15:1.

Projection Matrix and Its Size	CR	AVG. PRD	AVG. PRDN	QS
**PSCCS METHOD**				
Gaussian distribution Random * Dict ^†^	15:1	0.97	21.31	**15.46**
Bernoulli with 0 and 1 (20 × 300)	15:1	1.31	23.28	11.45
Gaussian distribution Random (20 × 300)	15:1	1.13	25.37	13.27
**CPCS METHOD**				
**Universal mega-dictionary without a centered R-wave**				
Gaussian distribution Random * Dict ^†^	15:1	0.88	13.67	**17.04**
Bernoulli with 0 and 1 (20 × 301)	15:1	1.44	21.43	10.41
Gaussian distribution Random (20 × 301)	15:1	1.62	24.33	9.25
**Universal mega-dictionary with a centered R-wave**				
Gaussian distribution Random * Dict ^†^	15:1	0.67	9.99	**22.38**
Bernoulli with 0 and 1 (20 × 301)	15:1	1.08	15.47	13.88
Gaussian distribution Random (20 × 301)	15:1	1.19	17.18	12.60
**Pathological specific dictionaries without a centered R-wave**				
Gaussian distribution Random * Dict ^†^	15:1	0.77	11.76	**19.48**
Bernoulli with 0 and 1 (20 × 301)	15:1	1.23	17.90	12.19
Gaussian distribution Random (20 × 301)	15:1	1.37	20.25	10.94
**Pathological specific dictionaries with a centered R-wave**				
Gaussian distribution Random * Dict ^†^	15:1	0.62	6.14	**24.19**
Bernoulli with 0 and 1 (20 × 301)	15:1	0.97	13.93	15.46
Gaussian distribution Random (20 × 301)	15:1	1.04	14.80	14.42
**Patient-specific dictionaries without a centered R-wave**				
Gaussian distribution Random * Dict ^†^	15:1	0.78	11.98	**19.23**
Bernoulli with 0 and 1 (20 × 301)	15:1	0.94	16.06	15.87
Gaussian distribution Random (20 × 301)	15:1	0.82	13.82	18.29
**Patient-specific dictionaries with a centered R-wave**				
Gaussian distribution Random * Dict ^†^	15:1	0.51	9	**29.13**
Bernoulli with 0 and 1 (20 × 301)	15:1	0.71	12.4	20.98
Gaussian distribution Random (20 × 301)	15:1	0.72	12.51	20.59

**Table 10:** Average values for 24 records and 117 record for other compression algorithms.

	Record/Ave.	CR	AVG. PRD	AVG. PRDN
Other Compression Algorithms				
Polania [20,21]	117	8:1	2.18	Notspec.
Polania [20,21]	117	10:1	2.5	Notspec.
Mamaghanian [22] for before and after inter-packet redundancy removal and Huffman coding	Ave. for 24 records	4:1 (75)	Before Huffman 35	
			After Huffman 15	
		10:1 (90)	Before Huffman >45	
			After Huffman >45	
		15:1 (93)	Before Huffman >45	
			After Huffman >45	

**Table 11:** Quality score for compression algorithms for average values for 24 records.

Algorithm	Average of Errors (PRD or RMS)	Average of CR	QS
Wavelet [23]	18.2 RMS	21.4:1	
SPHIT [24]	3.57 PRD	12:1	3.39
	4.85 PRD	16:1	3.29
	6.49 PRD	20:1	3.08
JPEG2000 [25]	2.19 PRD	12:1	5.47
	2.74 PRD	16:1	5.8
	3.26 PRD	20:1	6.1
QLV–Skeleton–Huffman * [26]	0.641 PRD *	16.9:1 *	29.36 *
Skeleton [10]	1.17 PRD 11.35 RMS	18.27:1	15.61
**PSCCS method**	**0.97 PRD**	**15:1**	**15.46**
**CS with patient-specific dictionaries with a centered R-wave**	**0.51 PRD**	**15:1**	**29.13**

NOTE: The results reported in [26] marked with * in Table 11 were obtained using a combined ECG compression method consisting of a preprocessing stage with quad level vector (QLV) for the extraction of the ECG skeleton achieving an 8.4:1 compression and a coding block (consisting of delta and Huffman Coding). The results referenced in Table 3 are the final one, improved by the Huffman coding stage.

## Data Availability

The data presented in this study are openly available in [physionet] at [10.1109/51.932724 and 10.1161/01.cir.101.23.e215], reference number [18]. The webpage of the MIT-BIH Arrhythmia Database is “https://www.physionet.org/content/mitdb/1.0.0/” (accessed on 27 December 2021).
